# Genome-wide DNA methylation association study of recent and cumulative marijuana use in middle aged adults

**DOI:** 10.1038/s41380-023-02106-y

**Published:** 2023-05-31

**Authors:** Drew R. Nannini, Yinan Zheng, Brian T. Joyce, Kyeezu Kim, Tao Gao, Jun Wang, David R. Jacobs, Pamela J. Schreiner, Kristine Yaffe, Philip Greenland, Donald M. Lloyd-Jones, Lifang Hou

**Affiliations:** 1 Department of Preventive Medicine, Northwestern University Feinberg School of Medicine, Chicago, IL USA; 2 Division of Epidemiology and Community Health, School of Public Health, University of Minnesota, Minneapolis, MN USA; 3 University of California at San Francisco School of Medicine, San Francisco, CA USA; 4 Department of Medicine, Northwestern University Feinberg School of Medicine, Chicago, IL USA

**Keywords:** Genetics, Biomarkers, Psychiatric disorders

## Abstract

Marijuana is a widely used psychoactive substance in the US and medical and recreational legalization has risen over the past decade. Despite the growing number of individuals using marijuana, studies investigating the association between epigenetic factors and recent and cumulative marijuana use remain limited. We therefore investigated the association between recent and cumulative marijuana use and DNA methylation levels. Participants from the Coronary Artery Risk Development in Young Adults Study with whole blood collected at examination years (Y) 15 and Y20 were randomly selected to undergo DNA methylation profiling at both timepoints using the Illumina MethylationEPIC BeadChip. Recent use of marijuana was queried at each examination and used to estimate cumulative marijuana use from Y0 to Y15 and Y20. At Y15 (*n* = 1023), we observed 22 and 31 methylation markers associated (FDR *P* ≤ 0.05) with recent and cumulative marijuana use and 132 and 16 methylation markers at Y20 (*n* = 883), respectively. We replicated 8 previously reported methylation markers associated with marijuana use. We further identified 640 *cis*-meQTLs and 198 DMRs associated with recent and cumulative use at Y15 and Y20. Differentially methylated genes were statistically overrepresented in pathways relating to cellular proliferation, hormone signaling, and infections as well as schizophrenia, bipolar disorder, and substance-related disorders. We identified numerous methylation markers, pathways, and diseases associated with recent and cumulative marijuana use in middle-aged adults, providing additional insight into the association between marijuana use and the epigenome. These results provide novel insights into the role marijuana has on the epigenome and related health conditions.

## Background

Marijuana is one of the most commonly used psychoactive substances in the US, with an estimated 49% of adults having ever used marijuana, including 19% within the past year, and 12% within the past month [1]. The prevalence of marijuana use has risen over the past several decades and its use is expected to increase as more states legalize marijuana [2–5]. Medically, marijuana may help treat chemotherapy induced nausea and vomiting [6], chronic neuropathic pain [7], inflammatory conditions [8, 9], Parkinson’s disease symptoms [10], and epilepsy [11]. Despite these therapeutic benefits, marijuana use may have adverse effects on health including short-term (e.g., impaired short-term memory and motor coordination, altered judgment, and psychotic symptoms) and long-term use (e.g., addiction, altered brain development, neurocognitive impairment, and cardiovascular and respiratory disease) [12, 13]. Additionally, marijuana use has been associated with increased risk of psychiatric disorders [14–16]. Due to the expected rise in use coinciding with legalization, studies investigating the association between marijuana use and molecular or epigenetic mechanisms may provide novel insights into the short- and long-term impacts of marijuana on health-related outcomes.

DNA methylation, one of the most-studied epigenetic modifications, is a regulatory process that affects gene expression (without altering the genomic sequence) through the addition or removal of methyl groups [17]. These modifications can be induced by environmental and lifestyle factors [18, 19], which may serve as blood-based biomarkers for recent and cumulative exposures. Additionally, the modifiable nature of DNA methylation allows for the investigation of exposure-induced changes to the epigenome and its variability across time, potentially leading to the identification of dynamic and/or stable biomarkers [20, 21]. These methylation changes may serve as biomarkers for recent and cumulative marijuana use, and subsequently, may further our understanding of the acute and additive influences of marijuana on molecular and biological processes influencing downstream health conditions.

Despite the growing use of marijuana, a limited number of studies have examined epigenome wide biomarkers associated with marijuana use. Previous studies have identified differentially methylated DNA signatures associated with marijuana, including markers located in *AHRR*, *ALPG*, *CEMIP*, and *MYO1G* [22, 23]. These biomarkers, however, were limited to a single time point and did not examine both recent and cumulative marijuana use. Studies examining the relationship between recent and cumulative marijuana use and epigenetic factors in a diverse population across time with repeated measurements may provide novel insights. Therefore, the purpose of this study was to investigate the association between recent and cumulative marijuana use and repeated genome-wide DNA methylation patterns measured in middle aged adults.

## Materials and methods

### Study population

The study design, recruitment, and follow-up of CARDIA were previously described [24]. Briefly, CARDIA is a population-based cohort study that recruited 5115 Black and White participants aged 18–30 from four centers across the US from 1985–1986. Participants were followed over time and underwent in-person examinations at baseline (year [Y] 0), Y2, Y5, Y7, Y10, Y15, Y20, Y25, Y30, and currently participating in Y35.

### Marijuana Use Measurements

At baseline (Y0) and each follow-up examination, study participants were asked “Have you ever used marijuana?”, “About how many times in your lifetime have you used marijuana?”, and “During the last 30 days, on how many days did you use marijuana?” For this analysis, we considered two continuous variables measuring recent and cumulative use of marijuana at both Y15 and Y20. For recent use, the number of days of marijuana use in the last 30 was used for analyses. For cumulative use, we calculated ‘marijuana-years’ from Y0 to Y15 and Y20 separately as previously described [25]. Assuming marijuana use in the last 30 days represents use throughout the year and between examinations, we summed the total number of days of marijuana use at Y0 to Y15 and Y20 separately and divided by 365 yielding marijuana-years, where a marijuana-year is equivalent to marijuana use once a day for a year.

### DNA methylation profiling

Details of blood sample collection and DNA processing have previously been described [26–28]. Briefly, a random sample of 1200 participants with available whole blood at both Y15 and Y20 underwent DNA methylation profiling using the Illumina MethylationEPIC BeadChip. Data process and quality control of the DNA methylation datasets were performed using the default settings in the R package Enmix [29]. Low quality methylation measurements were defined as markers with a detection *P* < 1E−06 or less than 3 beads. A total of 6209 markers with a detection rate <95% and 87 samples with methylation measurements of low-quality >5% or extremely low intensity of bisulfite conversion probes (defined as less than 3 times the standard deviation of the intensity across samples below the mean intensity) were removed from further analysis. Additionally, 95 samples were identified as extreme outliers as determined by the average total intensity value [intensity of unmethylated signals (U) + intensity of methylated signals (M)] or β value [M/(U + M + 100)] across all markers and Tukey’s method [30]. Model-based correction was applied using ENmix and dye bias correction was conducted using RELIC [31]. M or U intensities for Infinium I or II probes underwent quantile normalization separately, respectively. Low-quality methylation markers and β value outliers, as defined by Tukey’s method, were set to missing. After applying these criteria, 1042 and 957 samples at Y15 and Y20 remained for downstream analysis, respectively.

### Single time point and longitudinal analyses

We conducted single time point epigenome-wide association studies (EWASs) among CARDIA study participants with available DNA methylation and marijuana data at Y15 (*n* = 1023) and Y20 (*n* = 883). Linear regression was performed to analyze the association between DNA methylation levels for the 841,639 autosomal CpG sites modeled as the dependent variable and recent and cumulative marijuana use modeled as the independent variables at both timepoints (main EWAS). All models were adjusted for age, sex, self-reported race, study center, education, tobacco smoking status, physical activity, and alcohol consumption, as well as technical biases and leukocyte cell-type subpopulations. Principal component analysis was performed on intensity data for non-negative internal control probes and the top 8 principal components (PCs) were included as covariates. We used the Houseman’s method [32] to infer the proportion of leukocyte subpopulations (B cells, CD4 + T cells, CD8 + T cells, granulocytes, monocytes, and natural killer cells) and were included as covariates. Epigenomic control inflation factors [33] and quantile-quantile (Q-Q) plots were generated to assess for proper control of uncorrected technical biases and population stratification. CpG sites with a false-discovery rate (FDR) *P* value ≤ 0.05 at either Y15 or Y20 were considered statistically significant. We further investigated the longitudinal association between the change in marijuana use from Y15 to Y20 (Δmarijuana) and the change in methylation of marijuana associated CpGs from Y15 to Y20 (Δmethylation). Δmarijuana was estimated as the difference between recent and cumulative marijuana use at Y20 and Y15 and Δmethylation was estimated from the residuals in a linear model between Y20 and Y15 methylation levels, adjusted for the 8 PCs at both timepoints. The same linear regression EWAS model was performed with Δmethylation as the dependent variable and Δmarijuana as the independent variable, adjusting for the same covariates at both Y15 and Y20. All statistical analyses were performed using R 4.1.1 [34].

### Stratified analyses by sex, self-reported race, and tobacco smoking status

To further investigate observed recent and cumulative marijuana use CpGs, we performed stratified analyses at both timepoints by sex (Y15 [*n*_female_ = 521, *n*_male_ = 502] and Y20 [*n*_female_ = 453, *n*_male_ = 430]), self-reported race (Y15 [*n*_Black_ = 414, *n*_White_ = 609] and Y20 [*n*_Black_ = 366, *n*_White_ = 517]), and tobacco smoking status (Y15 [*n*_non_ = 644, *n*_former_ = 174, *n*_current_ = 205] and Y20 [*n*_non_ = 540, *n*_former_ = 171, *n*_current_ = 172]) for significant CpGs at Y15 and Y20. Models were adjusted for the same covariates as the main EWAS, except sex, self-reported race, and tobacco smoking status were excluded during the respective stratified analyses.

### Genotype imputation and methylation quantitative trait loci

To evaluate whether single nucleotide polymorphisms (SNPs) are associated with DNA methylation levels, we performed methylation quantitative trait loci (meQTL) analyses for significant recent and cumulative marijuana CpGs. Details on genotype imputation in CARDIA have previously been described [28]. Briefly, participants were genotyped using the Affymetrix Genome-Wide Human 6.0 array and untyped genotypes were imputed using the 1000 Genomes Project Phase 3 Integrated Release Version 5 reference panel using the programs SHAPEIT [35, 36] and Minimac3 [37]. After merging datasets, 182 and 160 Black participants and 485 and 408 White participants had both methylation and genotype data at Y15 and Y20, respectively. Analyses were performed separately by self-reported race at both examinations, adjusting for the same EWAS model covariates, using the program mach2qtl [38, 39]. We defined *cis*-meQTLs as SNPs within ±500,000 base pairs of the index CpG and *cis*-meQTLs with *P* value ≤ 2.82E−08 were considered statistically significant. Mapped trait information from NHGRI-EBI GWAS catalog was extracted and summarized for significant *cis*-meQTLs [40].

### Differentially methylated regions

To identify additional epigenetic loci associated with recent and cumulative marijuana use, we extended our analyses to examine differentially methylated regions (DMRs) using *comb-p* [41]. Previously, *comb-p* was found to have the highest sensitivity and control for false-positives compared to other DMR identification methods [42]. Analyses were ran using parameters previously identified to achieve the greatest performance, i.e., seed <0.05 and dist = 750 [42]. Associated DMRs were defined as having at least 3 probes and a Šidák corrected *P* value ≤ 0.05.

### Pathway and disease analyses

We performed pathway and disease analyses to examine the combined epigenetic associations of recent and cumulative marijuana use on pathways (KEGG and Reactome) and diseases (Disgenet, GLAD4U, and OMIM) using WebGestalt [43]. Due to the limited number of loci identified during single CpG analyses, probes were annotated to gene symbols according to the human genome assembly (hg19) [44] and the top 1000 annotated genes were included in overrepresentation enrichment analyses for recent and cumulative marijuana use at Y15 and Y20 separately. Pathways and diseases with an FDR *P* value ≤ 0.05 were considered statistically significant and the top five pathways and diseases were reported.

## Results

### Study characteristics

Table 1 presents descriptive characteristics for participants who underwent DNA methylation profiling at Y15 and Y20 by recent marijuana use. Among study participants, 71.9% and 70.1% reported having ever used marijuana and 13.7% and 12.8% reported using marijuana in the last 30 days at Y15 and Y20, respectively. Participants who recently used marijuana exhibited higher cumulative marijuana use at both Y15 and Y20 (*P* < 0.001), with an average ± standard deviation of 4.8 ± 3.8 and 6.1 ± 5.3 marijuana-years compared to 0.4 ± 0.9 and 0.5 ± 1.3 marijuana-years among those who did not recently use, respectively. Additionally, those who recently used marijuana were more likely to be current tobacco smokers compared to those who did not recently use, at both examination years (*P* < 0.001), i.e., 47.1% vs 15.7% at Y15 and 43.4% vs 16.0% at Y20.

**Table 1:** Study sample characteristics by recent marijuana use.

	Year 15			Year 20		
	No Recent Use	Recent Use	*P*	No Recent Use	Recent Use	*P*
*N*	883 (86.3)	140 (13.7)		770 (87.2)	113 (12.8)	
Female, *n* (%)	467 (52.9)	54 (38.6)	0.002	413 (53.6)	40 (35.4)	<0.001
Race, *n* (%)			0.122			0.061
Black	349 (39.5)	65 (46.4)		310 (40.3)	56 (50.0)	
White	534 (60.5)	75 (53.6)		460 (59.7)	57 (50.0)	
Age, mean (SD), years	40.4 (3.5)	40.5 (14.1)	0.716	45.4 (3.5)	45.4 (3.6)	0.887
Education, mean (SD), years	15.2 (2.5)	14.1 (2.4)	<0.001	15.1 (2.5)	14.3 (2.4)	0.002
Center, *n* (%)			0.004			<0.001
Birmingham, AL	228 (25.8)	23 (16.4)		192 (24.9)	17 (15.0)	
Chicago, IL	200 (22.6)	22 (15.7)		181 (23.5)	13 (11.5)	
Minneapolis, MN	224 (25.4)	50 (35.7)		190 (24.7)	42 (37.2)	
Oakland, CA	231 (26.2)	45 (32.2)		207 (26.9)	41 (36.3)	
Smoking status, *n* (%)			<0.001			<0.001
Nonsmoker	595 (67.4)	49 (35.0)		497 (64.5)	43 (38.0)	
Former Smoker	149 (16.9)	25 (17.9)		150 (19.5)	21 (18.6)	
Current Smoker	139 (15.7)	66 (47.1)		123 (16.0)	49 (43.4)	
Physical activity, mean (SD), intensity score	336.2 (270.1)	425.9 (288.7)	<0.001	337.9 (270.4)	418.5 (305.3)	0.009
Weekly alcoholic drinks, mean (SD), drinks	3.9 (7.3)	10.9 (15.6)	<0.001	4.1 (8.0)	11.5 (22.0)	<0.001
Day of marijuana use in last 30 days, mean (SD), days	0 (0.0)	11.1 (9.7)	<0.001	0 (0.0)	10.4 (9.9)	<0.001
Marijuana years, mean (SD), years	0.4 (0.9)	4.8 (3.8)	<0.001	0.5 (1.3)	6.1 (5.3)	<0.001

All statistics shown are mean and standard deviation (SD), except for number of participants, sex, self-reported race, center, smoking status, which are shown as number of participants and percentages.

Recent use is defined as use of marijuana within the last 30 days.

### Methylation markers of recent and cumulative marijuana use

Epigenomic control inflation factors were moderate (*λ* = 1.03–1.08) and inspection of the Q-Q plots (Supplementary Fig. 1) did not show deviation of the observed *P* values from the null, except at the extreme tails. These findings suggest proper control of technical biases and population stratification.

**Fig. 1:**
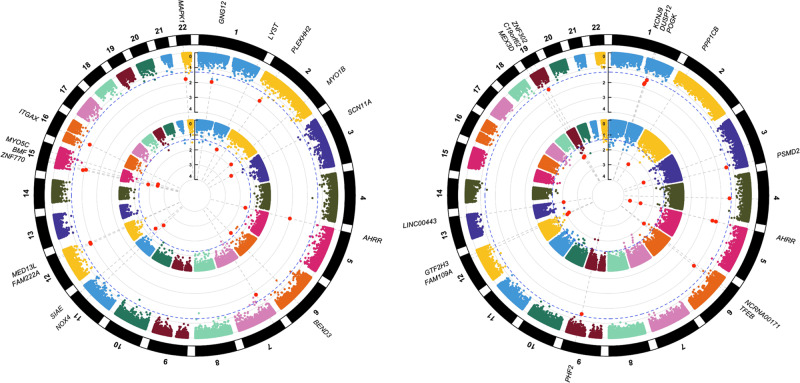
Circular Manhattan plots for CpGs associated with recent and cumulative marijuana use at Y15 and Y20. Recent and cumulative marijuana use association results correspond to the inner and outer circles at **A** Y15 and **B** Y20, respectively. The x-axis corresponds to epigenomic positions, and the y-axis shows the -log_10_ FDR. The horizontal dotted blue line denotes a significance threshold of FDR ≤ 0.05. The top 10 significant loci from each analysis are labeled in each plot.

Figure 1 displays circular Manhattan plots of the epigenome-wide FDR *P* values for recent and cumulative marijuana use at Y15 and Y20. In total, 201 methylation markers were associated (FDR *P* value ≤ 0.05) with marijuana across the two examination years. At Y15, recent and cumulative marijuana use were associated with 22 and 31 methylation markers, respectively (Supplementary Table 1). At Y20, recent and cumulative marijuana use were associated with 132 and 16 methylation markers, respectively (Supplementary Table 2). Intersection sets of recent and cumulative markers at Y15 identified 7 markers and 11 markers at Y20, with cg05575921 located in *AHRR* the only marker observed in all four analyses (Supplementary Fig. 2).

Table 2 summarizes the top 10 methylation markers for each analysis. Among the top CpGs, 6 were annotated to *AHRR*, including 3 out of the 4 most significant CpGs. Additional top loci associated with recent and cumulative marijuana use at Y15 include *MYO5C*, *SCN11A*, and *NOX4*, and *BMF*, *PLEKHH2*, and *FAM222A*, respectively. At Y20, *PP1CB*, *GTF2H3*, and *MEX3D*, and *TFEB*, *KCNJ9*, and *DUSP12* were top loci associated with recent and cumulative marijuana use, respectively.

**Table 2:** Top ten CpGs associated with recent and cumulative marijuana use at Y15 and Y20.

CpG	Chr	BP	Genomic Region	β	*P*	FDR	Gene
**Y15 Recent Use**							
cg18110140	15	75350380	–	−1.10E−02	7.39E−09	3.26E−03	–
cg21171274	15	52539081	Body	−3.81E−03	7.75E−09	3.26E−03	*MYO5C*
cg17218147	3	38995416	TSS1500	−5.11E−03	1.88E−08	5.26E−03	*SCN11A*
cg12438576	11	89232216	5’UTR	8.91E−03	3.28E−08	6.90E−03	*NOX4*
cg15627771	2	192111084	5’UTR	−4.91E−03	9.50E−08	1.42E−02	*MYO1B*
cg18880190	15	40399609	5’UTR	−5.01E−03	1.01E−07	1.42E−02	*BMF*
cg19857151	1	235985454	Body	5.66E−03	1.91E−07	2.29E−02	*LYST*
cg13444775	11	124546591	TSS1500	−1.29E−02	2.30E−07	2.42E−02	*SIAE*
cg05575921	5	373378	Body	−3.37E−02	3.38E−07	2.77E−02	*AHRR*
cg15607642	6	107391589	Body	5.62E−03	3.57E−07	2.77E−02	*BEND3*
**Y15 Cumulative Use**							
cg05575921	5	373378	Body	−8.51E−02	1.18E−09	9.93E−04	*AHRR*
cg18880190	15	40399609	5’UTR	−1.30E−02	2.00E−08	8.40E−03	*BMF*
cg07064251	2	43948676	Body	1.26E−02	6.89E−08	1.32E−02	*PLEKHH2*
cg23932689	12	110173904	5’UTR	1.52E−02	7.95E−08	1.32E−02	*FAM222A*
cg25189904	1	68299493	TSS1500	−3.81E−02	8.16E−08	1.32E−02	*GNG12*
cg13179084	12	116638602	Body	1.34E−02	9.46E−08	1.32E−02	*MED13L*
cg06326914	15	35280920	TSS1500	1.73E−02	1.10E−07	1.32E−02	*ZNF770*
cg12510044	22	22115473	3’UTR	−1.40E−02	1.82E−07	1.79E−02	*MAPK1*
cg18387338	7	26591438	–	−2.01E−02	2.08E−07	1.79E−02	–
cg04742550	16	31366429	TSS200	−2.98E−02	2.12E−07	1.79E−02	*ITGAX*
**Y20 Recent Use**							
cg21161138	5	399360	Body	−9.22E−03	3.33E−10	2.80E−04	*AHRR*
cg05575921	5	373378	Body	−3.41E−02	3.57E−09	1.50E−03	*AHRR*
cg06581729	2	29004756	Body	4.11E−05	9.61E−09	2.70E−03	*PPP1CB*
cg26867465	3	119399324	–	4.03E−03	1.40E−08	2.94E−03	–
cg01372788	12	124122424	5’UTR	1.04E−03	3.84E−08	6.47E−03	*GTF2H3*
cg19569686	19	1555141	3’UTR	1.18E−03	7.89E−08	9.25E−03	*MEX3D*
cg17456749	12	111807011	TSS200	−1.58E−03	8.06E−08	9.25E−03	*FAM109A*
cg00761236	13	107305783	TSS1500	−1.72E−03	8.80E−08	9.25E−03	*LINC00443*
cg09607178	6	29978432	Body	1.95E−03	1.06E−07	9.90E−03	*NCRNA00171*
cg02337960	19	17378645	5’UTR	−7.34E−04	1.66E−07	1.03E−02	*C19orf62*
**Y20 Cumulative Use**							
cg05575921	5	373378	Body	−6.94E−02	5.00E−09	4.21E−03	*AHRR*
cg21161138	5	399360	Body	−2.15E−02	2.17E−08	7.37E−03	*AHRR*
cg09040721	6	41658881	Body	5.42E−03	2.63E−08	7.37E−03	*TFEB*
cg13552867	1	160053689	5’UTR	−9.97E−03	8.81E−08	1.85E−02	*KCNJ9*
cg09825346	1	161718614	TSS1500	1.23E−02	1.34E−07	2.25E−02	*DUSP12*
cg21263605	1	166818686	Body	6.78E−04	3.75E−07	3.76E−02	*POGK*
cg26003997	9	96395832	Body	−1.23E−03	4.13E−07	3.76E−02	*PHF2*
cg24976193	4	100685228	–	−1.22E−02	4.24E−07	3.76E−02	–
cg00785657	19	35168593	1stExon	1.38E−03	4.24E−07	3.76E−02	*ZNF302*
cg02646643	3	184026751	3’UTR	2.24E−03	4.71E−07	3.76E−02	*PSMD2*

Beta coefficient represents the change in DNA methylation level (i.e., M value) for each additional day of recent marijuana use or additional marijuana year.

Results are adjusted for age, sex, self-reported race, study center, education, tobacco smoking status, physical activity, and alcohol consumption, PC1-PC8, and leukocyte cell-type subpopulations.

### ΔMarijuana use vs. Δmethylation analysis

Of the 22 and 132 methylation markers associated with recent marijuana use at Y15 and Y20, 13 and 124 markers yielded consistent direction of associations during Δmethylation and Δmarijuana analyses (*r* = 0.756; *P* = 4.72E−05 and *r* = 0.861; *P* < 2.20E−16, respectively; Supplementary Table 3; Supplementary Fig. 3A). Of the 31 and 16 methylation markers associated with cumulative marijuana use at Y15 and Y20, 20 and 16 markers yielded consistent direction of associations during Δmethylation and Δmarijuana analyses (*r* = 0.679; *P* = 2.69E−05 and *r* = 0.933; *P* = 1.39E−07, respectively; Supplementary Table 4; Supplementary Fig. 3B).

### Stratified analysis by sex

At Y15, 17 and 50 markers of the 53 total identified markers remained associated among Female and Male participants, respectively (Supplementary Table 5). The Y15 regression coefficients for recent and cumulative marijuana use were highly correlated between Female and Male participants (*r* = 0.945; *P* = 3.82E−11 and *r* = 0.975; *P* < 2.20E−16) (Supplementary Fig. 4). At Y20, 26 and 112 markers of the 148 total identified markers remained associated among Female and Male participants, respectively (Supplementary Table 6). The regression coefficients between Female and Male participants were highly correlated (*r* = 0.846; *P* < 2.20E−16 and *r* = 0.952; *P* = 1.41E−08) (Supplementary Fig. 5).

### Stratified analysis by self-reported race

Of the 53 total identified methylation markers at Y15, 26 and 48 markers remained associated among Black and White participants, respectively (Supplementary Table 7). Additionally, the regression coefficients for recent and cumulative marijuana use at Y15 were highly correlated between Black and White participants (*r* = 0.950; *P* = 1.40E−11 and *r* = 0.928; *P* = 6.27E−14) (Supplementary Fig. 6). Of the 148 total identified methylation markers at Y20, 65 and 64 markers remained associated among Black and White participants, respectively (Supplementary Table 8). The regression coefficients between Black and White participants were highly correlated (*r* = 0.930; *P* < 2.20E−16 and *r* = 0.959; *P* = 4.44E−09) (Supplementary Fig. 7).

### Stratified analysis by tobacco smoking status

To determine whether associations of identified CpGs differed by tobacco smoking status, we investigated these CpGs by strata of tobacco use. At Y15, 25, 18, and 20 markers of the 53 total identified markers remained associated among nonsmokers, former smokers, and current smokers, respectively (Supplementary Table 9). The regression coefficients for recent and cumulative marijuana use at Y15 were highly correlated across tobacco smoking status (*r* range: 0.837–0.939) (Supplementary Figs. 8, 9). At Y20, 51, 19, and 26 markers of the 148 total markers remained associated among nonsmokers, former smokers, and current smokers, respectively (Supplementary Table 10). The regression coefficients were highly correlated across tobacco smoking status at Y20 (*r* range: 0.730–0.934) (Supplementary Figs. 10, 11).

### Replication of previously reported marijuana CpGs

We evaluated the associations of previously reported marijuana methylation markers. Overall, 31 CpGs were identified from previous studies (Supplementary Tables 11–12). After applying a Bonferroni correction (0.05/31 = 0.0016), 8 and 6 CpGs were associated with recent marijuana use and 8 and 7 CpGs were associated with cumulative marijuana use at Y15 and Y20, respectively, including markers in *AHRR*, *MYO1G*, *ALPG*, *F2RL3*, and *RARA*.

### Cis-methylation quantitative trait loci analyses and GWAS catalog mapping

To determine whether genetic markers influence methylation levels, we examined SNPs within ±500,000 base pairs of the identified CpGs. A total of 27 and 350 *cis*-meQTLs were associated with recent and cumulative marijuana use in Black and White participants at Y15, respectively (Supplementary Table 13). Specifically, 27 *cis*-meQTLs were associated with cg18110140 among Black participants and 345 and 5 *cis*-meQTLs were associated with cg18110140 and cg18880190 among White participants, respectively. Additionally, 261 *cis*-meQTLs were associated with cg19414984 for recent marijuana use at Y20 among White participants (Supplementary Table 14). Mapping these *cis*-meQTLs to the NHGRI-EBI GWAS Catalog identified 120 unique traits for 71 *cis*-meQTLs including blood pressure, brain measurements, coffee consumption, cortical surface area, immunological factors, multisite chronic pain, self-reported educational attainment, and smoking status (Supplementary Table 15).

### Differentially methylated regional analysis

To pursue additional epigenomic regions not identified during single marker analysis, we performed DMR analyses for recent and cumulative marijuana use at Y15 and Y20. A total of 47 and 54 DMRs were observed to be associated with recent and cumulative marijuana use at Y15, respectively (Supplementary Tables 16, 17). Additionally, 53 and 44 DMRs were associated with recent and cumulative marijuana use at Y20, respectively (Supplementary Tables 18, 19). DMRs annotated to the nearest gene identified 8 overlapping genes for both recent and cumulative marijuana use at Y15 and Y20. An intersection set of all DMRs identified 6 loci: *GNG12-AS1*, *HOXB-AS3*, *MYO1G*, *RNF39*, *SDHAP3*, and *ZNF578*.

### Pathway and disease analyses

Table 3 presents the top 5 KEGG and Reactome pathways from WebGestalt statistically associated with recent and cumulative marijuana use at Y15 and Y20. At Y15, the top pathways associated with recent marijuana use are related to MAPK signaling, diseases of signal transduction, and the neuronal system; the top pathways associated with cumulative use include Rho GTPase, cell proliferation and apoptosis, and depolarization. At Y20, the top pathways associated with recent marijuana use are related to dopamine synapses, diseases of signal transduction, transcription, human papillomavirus infection, and oxytocin signaling; the top pathways associated with cumulative use include diseases of signal transduction, transcription regulation by RUNX2, WNT signaling, human papillomavirus infection, and oxytocin signaling.

**Table 3:** Top five KEGG and Reactome pathways associated with recent and cumulative marijuana use at Y15 and Y20.

Pathway	Observed/Total Genes	*P*	FDR
**Y15 Recent Use**			
MAPK Family Signaling Cascades	28/293	2.37E−07	5.05E−04
MAPK1/MAPK3 Signaling	25/254	6.02E−07	8.55E−04
RAF/MAP Kinase Cascade	24/248	1.36E−06	1.29E−03
Diseases of Signal Transduction	30/378	4.41E−06	3.75E−03
Neuronal System	29/368	7.27E−06	5.63E−03
**Y15 Cumulative Use**			
Signaling by Rho GTPases	36/444	3.73E−07	1.27E−03
Rho GTPase Cycle	18/138	4.49E−07	1.27E−03
G alpha (12/13) Signaling Events	13/79	1.25E−06	2.59E−03
Phase 0—Rapid Depolarization	10/46	1.52E−06	2.59E−03
NRAGE Signals Death through JNK	11/59	2.31E−06	3.27E−03
**Y20 Recent Use**			
Dopaminergic Synapse	18/131	1.91E−07	5.43E−04
Diseases of Signal Transduction	32/378	5.93E−07	1.26E−03
Generic Transcription Pathway	68/1169	1.27E−06	1.80E−03
Human Papillomavirus Infection	29/339	1.61E−06	1.89E−03
Oxytocin Signaling Pathway	18/152	1.78E−06	1.89E−03
**Y20 Cumulative Use**			
Diseases of Signal Transduction	36/378	5.57E−09	3.72E−05
Transcriptional Regulation by RUNX3	17/96	8.74E−09	3.72E−05
Beta-Catenin Independent WNT Signaling	20/145	3.70E−08	1.03E−04
Human Papillomavirus Infection	32/339	4.85E−08	1.03E−04
Oxytocin Signaling Pathway	20/152	8.24E−08	1.40E−04

Table 4 presents the top 5 diseases statistically associated with recent and cumulative marijuana use at both examination years. At Y15, the top diseases associated with recent marijuana include schizophrenia, mental disorders, bipolar disorder, and substance-related disorders; the top diseases associated with cumulative use include disease susceptibility, mental disorders, autistic disorder, and genetic predisposition to disease. At Y20, top diseases associated with recent marijuana use include mental disorders, schizophrenia, short stature, brachydactyly, and spastic tetraparesis and the top diseases associated with cumulative marijuana use include schizophrenia, drug-drug interaction, genetic predisposition to disease, disease susceptibility, and liver cirrhosis.

**Table 4:** Top five diseases associated with recent and cumulative marijuana use at Y15 and Y20.

Disease	Observed/Total Genes	*P*	FDR
**Y15 Recent Use**			
Schizophrenia	67/1041	3.17E−08	2.20E−04
Mental Disorders	53/757	6.70E−08	2.20E−04
Bipolar Disorder	41/516	7.75E−08	2.20E−04
Adhesion	51/766	5.52E−07	8.55E−04
Substance-Related Disorders	16/115	7.12E−07	8.66E−04
**Y15 Cumulative Use**			
Disease Susceptibility	61/970	4.16E−07	1.27E−03
Mental Disorders	48/757	5.90E−06	6.67E−03
Autistic Disorder	23/252	7.05E−06	6.67E−03
Genetic Predisposition to Disease	57/966	7.05E−06	6.67E−03
Adhesion	48/766	8.08E−06	6.88E−03
**Y20 Recent Use**			
Mental Disorders	57/757	1.91E−09	1.63E−05
Schizophrenia	67/1041	3.91E−08	1.66E−04
Short Stature	40/545	1.00E−06	1.70E−03
Brachydactyly	15/125	1.08E−05	6.85E−03
Spastic Tetraparesis	5/12	2.13E−05	7.73E−03
**Y20 Cumulative Use**			
Schizophrenia	62/1041	1.71E−06	7.80E−04
Drug Interaction with Drug	35/494	9.89E−06	2.48E−03
Genetic Predisposition to Disease	55/966	2.39E−05	4.15E−03
Disease Susceptibility	55/970	2.68E−05	4.38E−03
Liver Cirrhosis, Experimental	44/767	1.32E−04	1.46E−02

## Discussion

In this multiple timepoint epigenome-wide association study of middle-aged adults, we observed 201 methylation markers associated with recent and cumulative marijuana use across time. We replicated 8 previously reported methylation markers associated with marijuana use. We also observed 638 *cis*-meQTLs associated with several marijuana-methylation markers, as well as 198 differentially methylated regions. During pathway and disease analyses, marijuana-associated genes were statistically overrepresented in numerous pathways and diseases. While replication of these findings in independent cohorts is warranted, our results provide novel insights into the association between recent and cumulative marijuana use and the epigenome and related biological processes, which may serve as a mechanism of early-stage disease associated with marijuana use.

We identified numerous methylation markers associated with recent and cumulative marijuana use. Of these, cg05575921 in *AHRR* was associated with recent and cumulative marijuana use at both timepoints, including the single most-associated methylation marker for two of the four analyses. This methylation marker has previously been associated with heavy cannabis use among tobacco users [22], tobacco use [45–47], and is 1 of 172 CpGs included in the estimation of a DNA methylation surrogate for pack-years of smoking (DNAmPACKYRS) for GrimAge, a measure of biological age associated with lifespan [48]. The association of this epigenetic marker with both tobacco and marijuana use may suggest common modulating effects on DNA methylation and may represent a nondiscriminatory smoke related biomarker, irrespective of tobacco or marijuana use. Additionally, cg05575921 has been associated with psychiatric disorders [49, 50]. The top methylation marker associated with recent marijuana use at Y15, cg18110140, is located on chromosome 15 in an ‘open sea’ region of the epigenome. This marker was recently found to be associated with smoking status [51–53]. Several top epigenomic loci have also previously been associated with tobacco smoking, including *BMF* and *MYO1B* [52], and may provide additional measurable biomarkers for tobacco and marijuana exposure. Moreover, numerous epigenomic loci have been reported to have potential therapeutic benefits via the endocannabinoid system. *NOX4* is a member of the NADPH oxidase family and an enzyme that synthesizes reactive oxygen species (ROS) and cannabidiol (CBD), one of the most common cannabinoids, has been reported to attenuate ROS formation and enhance expression of NOX4 [54]. Similarly, *TFEB* is associated with the autophagy-lysosomal pathway and may aid in reducing inflammation and cognitive impairment via the cannabinoid receptor type II [55]. Although the effect estimates for the observed associations are relatively small, the magnitude of the beta coefficients are consistent with previous EWAS studies [22, 28] and further studies investigating the cumulative effect of these individual CpGs (e.g., polyepigenetic risk score) may yield greater biological, and potentially clinical, relevance. We also replicated several previously reported marijuana loci, i.e., *AHRR*, *ALPG*, *F2RL3*, and *MYO1G* [22], in this mixed sex and self-reported race study sample, although additional studies in more diverse populations are needed to further evaluate previously associated epigenetic markers. Additionally, we observed differential DNA methylation levels by self-reported race and tobacco smoking status. While regression coefficients were highly correlated during stratified analyses, these findings provide insight into the interactive roles of self-reported race and tobacco smoking on marijuana associated methylation markers. For example, recent and cumulative marijuana use tended to exhibit greater hypomethylation of cg05575921 among Black participants and nonsmokers compared to White participants and former and current smokers, respectively. For the latter finding, the hypomethylation of cg05575921 during pooled and stratified analyses by tobacco smoking status suggests marijuana’s association with methylation may be consistent and independent of tobacco smoking. Our results highlight the interactive influences of biological and environmental factors on methylation signatures and provide insight into the differing impact of marijuana on the epigenome by population strata. These findings may serve as potential biomarkers to identify recent and long-term marijuana use and molecular targets for further investigation.

The epigenome is dynamic and responsive to environmental and lifestyle factors throughout the lifespan. Due to the ever-changing nature of the epigenome, evaluating differences in methylation patterns across time not only enables the temporal (and, potentially, causal) assessment of a phenotype and epigenetic changes in the context of the natural history of a disease, but also permits examination of intra- and inter-individual variability and trajectories in methylation patterns over time [56]. Additionally, longitudinal epigenetic studies will allow for the examination of the impact of interventions on epigenetic changes. For example, longitudinal examination of smoking-induced DNA methylation patterns identified dynamic and stable markers across time and also observed reversal of smoking induced methylation changes after smoking cessation [57, 58]. Using repeated measures of DNA methylation and marijuana use, we cross-sectionally identified numerous marijuana associated epigenetic markers associated at one time point but not the other (i.e., dynamic), including 6 (e.g., *BEND3* and *GNG12*) and 10 (e.g., *PHF2* and *PSMD2*) loci associated with both recent and cumulative marijuana use at Y15 and Y20, respectively. Additionally, one stable epigenetic marker, cg05575921, was associated with both marijuana variables across the examination years with consistent effect estimates (recent use: β_Y15_ = −3.37E−02 vs β_Y20_ = −3.41E−02; cumulative use: β_Y15_ = −8.51E−02 vs β_Y20_ = −6.94E−02). We also performed longitudinal analyses to investigate changes in methylation and marijuana use across the examinations and identified 12 CpGs that varied with change in marijuana use, including markers in *AHRR*, *COL11A2*, and *TFEB*. Together, these results suggest a majority of the observed marijuana associated epigenetic associations are dynamic, although stable epigenetic patterns maybe observed with marijuana use. Furthermore, the identification of dynamic markers across time suggests both recent and cumulative marijuana use may modulate epigenetic changes differently during the aging process. A possible explanation for the observation of different CpGs, as well as biological pathways and diseases, across the timepoints may relate to the pharmacokinetic properties influenced by age. For example, reductions in hepatic and renal clearance can increase the bioavailability of marijuana metabolites with prolongation of its half-life and subsequently, may impact molecular and cellular processes differently by age [59]. Consistent with our findings, dynamic epigenetic markers are more likely to be identified compared to stable markers during longitudinal analyses [60]. However, further studies investigating the modulatory effects of marijuana on the epigenome on different age groups may provide additional insight. Moreover, changes in marijuana use may alter DNA methylation signatures, which may serve as biomarkers to evaluate continued or ceased marijuana use. Although additional studies are needed to evaluate these markers, our findings demonstrate marijuana may induce dynamic and stable epigenetic signatures that may have utility as biomarkers for recent and cumulative marijuana use across time.

The impact of lifestyle factors and behaviors on health is complex and often involves an integrative approach to elucidate the underlying biological processes. By investigating genetic contributions to methylation markers associated with marijuana use, we identified 650 *cis*-meQTLs, including 56 *cis*-meQTLs that mapped to traits in the NHGRI-EBI GWAS Catalog. Among the mapped traits, we observed consistent terms related to immunological factors, cardiovascular traits, and brain measurements. Marijuana use has been associated with alterations in white blood cell counts [61], blood pressure [62], and brain structures [63]. We found the most significant mapped *cis*-meQTL has previously been associated with coffee consumption. Caffeine is the most consumed psychoactive substance in the world and induces dopamine release in the nucleus accumbens, a brain structure mediating pleasure and reward processing [64]. Analogously, marijuana exerts similar effects on the nucleus accumbens via the endocannabinoid system [65], suggesting the pleasure and reward of caffeine and marijuana use share the same reward center. Additionally, we identified 198 DMRs associated with recent and cumulative use of marijuana at Y15 and Y20. Among the top DMRs, several regions have previously been associated with cognitive function, psychiatric disorders, and immune function. *RNF39* was the most significant DMR in two of the four analyses and has previously been associated with general cognitive function [66] and bipolar and major depressive disorders [67]. *TRIOBP* is the most significant DMR associated with recent marijuana use at Y20 and has been associated with general cognitive function [66, 68], schizophrenia [69], and basophil count [70]. Similarly, *SH3RF3* has been associated with general cognitive ability [66], schizophrenia [71], and eosinophilia [72]. Lastly Z*FP57* has been associated with general cognitive ability [66, 68], schizophrenia [73], autism [74], and rheumatoid arthritis [75]. In sum, these findings suggest marijuana use shares common genetic and epigenetic pathways associated with immunological factors, cognitive function, and brain structures and may regulate similar molecular mechanisms and biological processes. These insights could help lead to the development of new preventive and predictive tools for marijuana-associated health outcomes.

As a psychoactive substance, marijuana may modulate pathways and diseases associated with homeostasis and health outcomes. Our pathway analysis revealed differentially methylated markers overrepresented in pathways associated with cellular proliferation, hormone signaling, and infection. The MAPK signaling cascades are signaling pathways that regulate cellular proliferation, differentiation, and apoptosis; studies have suggested potential therapeutic benefits of CBD on cancer treatment via these pathways [76, 77]. With regard to hormones, the endocannabinoid system modulates dopaminergic neurons and acute use of tetrahydrocannabinol (THC) increases dopamine release and neuron activity, whereas long-term use has been associated with diminishing of the dopamine system [78]. THC has also been shown to modulate oxytocin and areas of the brain associated with reward and addiction behaviors [79]. Moreover, cannabinoids have been reported to promote progression of human papillomavirus positive head and neck squamous cell carcinoma, primarily through MAPK activation [80]. Notably, a previous genome-wide DNA methylation study of marijuana identified the latter two pathways during pathway analysis [22]. In addition to these biological pathways, differentially methylated genes associated with marijuana use were overrepresented in psychiatric diseases and spasticity. Marijuana use has been associated with several psychotic disorders including schizophrenia [81, 82], bipolar disorder [83, 84], autism [85], and psychosis [86], as well as substance-related disorders [87, 88]. Additionally THC [89] and smoked marijuana [90] have been shown to reduce spasticity among patients with multiple sclerosis and spinal cord injuries. Additionally, connections between the top marijuana associated pathways and diseases have been previously reported. For example, abnormalities in the MAPK signaling [91] and dopamine pathways [92] have been associated with schizophrenia, as well as the use of oxytocin for treatment of substance related disorders [93]. Collectively, we identified pathways and diseases overrepresented with marijuana-associated methylation markers, suggesting common epigenetic regulations which could serve as potential diagnostic and therapeutic targets for these related traits.

The current CARDIA study leveraged repeated methylation levels and marijuana data to examine the association of marijuana use on DNA methylation. The availability of genetic data enabled the examination of potential genetic modulation of methylation markers associated with marijuana via meQTL analyses. Moreover, compared to other countries where residents use a mixture of marijuana and tobacco, CARDIA is a US-based cohort where mixing of marijuana and tobacco is less prevalent, allowing for a more complete examination of the independent associations of marijuana and tobacco smoking on DNA methylation [94]. This study, however, is not without limitations. Although we identified biologically relevant epigenetic loci and replicated previously reported methylation markers, we were unable to replicate our findings in an independent study, and as such, the findings presented warrant validation. Residual confounding from additional factors, e.g., use of other or co-drug use and social support, may partially explain the observed associations. As marijuana use was considered illegal for most yearly examinations in CARDIA, use may have been underreported. However, at each examination, marijuana use was self-reported (as opposed to interviewer obtained), collected at a research site (rather than an employer), and participants’ responses were confidential [95]. The route of administration of marijuana can also affect the onset, intensity, and duration of the psychoactive effects, as well as organ systems [96]. Investigations into marijuana use via other routes of administration (e.g., edibles, pills, vaping) may provide novel additional insights, including the latter, which was not present during the timepoints in the current study but is becoming more widely used. Additionally, this study examined acute exposure to marijuana (within the last 30 days), compared to hyperacute exposure (within hours) and investigations into DNA methylation changes due to hyperacute exposure may provide further insight into the acuity of exposure on epigenetic factors. And lastly, although CARDIA is a diverse cohort, Black and White participants were sampled from four centers across the US. As such, additional studies from more diverse populations across different geographical locations will enable for better generalizability of the findings presented here.

## Conclusion

In conclusion, we observed significant associations between recent and cumulative marijuana use with DNA methylation markers across time. We also observed *cis*-meQTLs and DMRs associated with marijuana use and biologically relevant pathways and diseases, suggesting potential shared genes between marijuana use and cellular proliferation, hormone signaling, and mental disorders. Additional studies are needed to replicate and verify the observed associations presented here. With the greater number of states legalizing marijuana for medical and recreational use, as well as the expected rise in its use, examining the association between marijuana and the epigenome may aid in elucidating the molecular and biological processes influencing downstream health conditions and may serve as potential biomarkers to identify recent and long-term marijuana use and intervene in the early stages of their related health outcomes.

### Supplementary information

Supplemental Figures

Supplemental Tables

## Data Availability

Data are available from the corresponding author on reasonable request.

## References

[1] Substance Abuse and Mental Health Services Administration (SAMHSA). 2018 National Survey on Drug Use and Health (NSDUH): Table 1.3B—Types of Illicit Drug Use in Lifetime, Past Year, and Past Month among Persons Aged 18 or Older: Percentages, 2017 and 2018. Substance Abuse and Mental Health Services Administration, U.S.: Department of Health & Human Services; 2018.

[2] Hasin DS, Saha TD, Kerridge BT, Goldstein RB, Chou SP, Zhang H (2015). Prevalence of Marijuana Use Disorders in the United States Between 2001-2002 and 2012-2013. JAMA Psychiatry.

[3] Yu B, Chen X, Chen X, Yan H (2020). Marijuana legalization and historical trends in marijuana use among US residents aged 12-25: results from the 1979-2016 National Survey on drug use and health. BMC Public Health.

[4] Cerda M, Mauro C, Hamilton A, Levy NS, Santaella-Tenorio J, Hasin D (2020). Association Between Recreational Marijuana Legalization in the United States and Changes in Marijuana Use and Cannabis Use Disorder From 2008 to 2016. JAMA Psychiatry.

[5] Mauro CM, Newswanger P, Santaella-Tenorio J, Mauro PM, Carliner H, Martins SS (2019). Impact of Medical Marijuana Laws on State-Level Marijuana Use by Age and Gender, 2004-2013. Prev Sci.

[6] Smith LA, Azariah F, Lavender VT, Stoner NS, Bettiol S. Cannabinoids for nausea and vomiting in adults with cancer receiving chemotherapy. Cochrane Database Syst Rev. 2015. 10.1002/14651858.CD009464.pub2. 10.1002/14651858.CD009464.pub2 PMC6931414 26561338

[7] Ware MA, Wang T, Shapiro S, Robinson A, Ducruet T, Huynh T (2010). Smoked cannabis for chronic neuropathic pain: a randomized controlled trial. CMAJ.

[8] Nagarkatti P, Pandey R, Rieder SA, Hegde VL, Nagarkatti M (2009). Cannabinoids as novel anti-inflammatory drugs. Future Med Chem.

[9] Esposito G, Filippis DD, Cirillo C, Iuvone T, Capoccia E, Scuderi C (2013). Cannabidiol in inflammatory bowel diseases: a brief overview. Phytother Res.

[10] Lotan I, Treves TA, Roditi Y, Djaldetti R (2014). Cannabis (medical marijuana) treatment for motor and non-motor symptoms of Parkinson disease: an open-label observational study. Clin Neuropharmacol.

[11] O’Connell BK, Gloss D, Devinsky O (2017). Cannabinoids in treatment-resistant epilepsy: A review. Epilepsy Behav.

[12] Volkow ND, Baler RD, Compton WM, Weiss SR (2014). Adverse health effects of marijuana use. N Engl J Med.

[13] Karila L, Roux P, Rolland B, Benyamina A, Reynaud M, Aubin HJ (2014). Acute and long-term effects of cannabis use: a review. Curr Pharm Des.

[14] Moore TH, Zammit S, Lingford-Hughes A, Barnes TR, Jones PB, Burke M (2007). Cannabis use and risk of psychotic or affective mental health outcomes: a systematic review. Lancet.

[15] Gage SH, Hickman M, Zammit S (2016). Association Between Cannabis and Psychosis: Epidemiologic Evidence. Biol Psychiatry.

[16] Di Forti M, Quattrone D, Freeman TP, Tripoli G, Gayer-Anderson C, Quigley H (2019). The contribution of cannabis use to variation in the incidence of psychotic disorder across Europe (EU-GEI): a multicentre case-control study. Lancet Psychiatry.

[17] Dor Y, Cedar H (2018). Principles of DNA methylation and their implications for biology and medicine. Lancet.

[18] Alegria-Torres JA, Baccarelli A, Bollati V (2011). Epigenetics and lifestyle. Epigenomics.

[19] Ronn T, Volkov P, Davegardh C, Dayeh T, Hall E, Olsson AH (2013). A six months exercise intervention influences the genome-wide DNA methylation pattern in human adipose tissue. PLoS Genet.

[20] Guida F, Sandanger TM, Castagne R, Campanella G, Polidoro S, Palli D (2015). Dynamics of smoking-induced genome-wide methylation changes with time since smoking cessation. Hum Mol Genet.

[21] Dugue PA, Wilson R, Lehne B, Jayasekara H, Wang X, Jung CH (2021). Alcohol consumption is associated with widespread changes in blood DNA methylation: Analysis of cross-sectional and longitudinal data. Addict Biol.

[22] Osborne AJ, Pearson JF, Noble AJ, Gemmell NJ, Horwood LJ, Boden JM (2020). Genome-wide DNA methylation analysis of heavy cannabis exposure in a New Zealand longitudinal cohort. Transl Psychiatry.

[23] Markunas CA, Hancock DB, Xu Z, Quach BC, Fang F, Sandler DP (2021). Epigenome-wide analysis uncovers a blood-based DNA methylation biomarker of lifetime cannabis use. Am J Med Genet B Neuropsychiatr Genet.

[24] Friedman GD, Cutter GR, Donahue RP, Hughes GH, Hulley SB, Jacobs DR (1988). CARDIA: study design, recruitment, and some characteristics of the examined subjects. J Clin Epidemiol.

[25] Pletcher MJ, Vittinghoff E, Kalhan R, Richman J, Safford M, Sidney S (2012). Association between marijuana exposure and pulmonary function over 20 years. JAMA.

[26] Joyce BT, Gao T, Zheng Y, Ma J, Hwang SJ, Liu L (2021). Epigenetic Age Acceleration Reflects Long-Term Cardiovascular Health. Circ Res.

[27] Nannini DR, Joyce BT, Zheng Y, Gao T, Liu L, Yoon G (2019). Epigenetic age acceleration and metabolic syndrome in the coronary artery risk development in young adults study. Clin Epigenetics.

[28] Zheng Y, Joyce B, Hwang SJ, Ma J, Liu L, Allen N, et al. Association of Cardiovascular Health Through Young Adulthood With Genome-Wide DNA Methylation Patterns in Midlife: The CARDIA Study. Circulation. 2022. 10.1161/CIRCULATIONAHA.121.055484. 10.1161/CIRCULATIONAHA.121.055484 PMC9348746 35652342

[29] Xu Z, Niu L, Li L, Taylor JA (2016). ENmix: a novel background correction method for Illumina HumanMethylation450 BeadChip. Nucleic Acids Res.

[30] Tukey J. Exploratory Data Analysis. Pearson; Massachusetts, United States: Addison-Wesley Publishing Company; 1977.

[31] Xu Z, Langie SA, De Boever P, Taylor JA, Niu L (2017). RELIC: a novel dye-bias correction method for Illumina Methylation BeadChip. BMC Genomics.

[32] Houseman EA, Accomando WP, Koestler DC, Christensen BC, Marsit CJ, Nelson HH (2012). DNA methylation arrays as surrogate measures of cell mixture distribution. BMC Bioinformatics.

[33] van Iterson M, van Zwet EW, Heijmans BT, Consortium B (2017). Controlling bias and inflation in epigenome- and transcriptome-wide association studies using the empirical null distribution. Genome Biol.

[34] R Core Team. R: A language and environment for statistical computing. R Foundation for Statistical Computing: Vienna, Austria, (2020).

[35] Delaneau O, Marchini J, Zagury JF (2011). A linear complexity phasing method for thousands of genomes. Nat Methods.

[36] Delaneau O, Zagury JF, Marchini J (2013). Improved whole-chromosome phasing for disease and population genetic studies. Nat Methods.

[37] Das S, Forer L, Schonherr S, Sidore C, Locke AE, Kwong A (2016). Next-generation genotype imputation service and methods. Nat Genet.

[38] Li Y, Willer C, Sanna S, Abecasis G (2009). Genotype imputation. Annu Rev Genomics Hum Genet.

[39] Li Y, Willer CJ, Ding J, Scheet P, Abecasis GR (2010). MaCH: using sequence and genotype data to estimate haplotypes and unobserved genotypes. Genet Epidemiol.

[40] Buniello A, MacArthur JAL, Cerezo M, Harris LW, Hayhurst J, Malangone C (2019). The NHGRI-EBI GWAS Catalog of published genome-wide association studies, targeted arrays and summary statistics 2019. Nucleic Acids Res.

[41] Pedersen BS, Schwartz DA, Yang IV, Kechris KJ (2012). Comb-p: software for combining, analyzing, grouping and correcting spatially correlated P-values. Bioinformatics.

[42] Mallik S, Odom GJ, Gao Z, Gomez L, Chen X, Wang L (2019). An evaluation of supervised methods for identifying differentially methylated regions in Illumina methylation arrays. Brief Bioinform.

[43] Liao Y, Wang J, Jaehnig EJ, Shi Z, Zhang B (2019). WebGestalt 2019: gene set analysis toolkit with revamped UIs and APIs. Nucleic Acids Res.

[44] Hansen K. IlluminaHumanMethylationEPICanno.ilm10b2.hg19: Annotation for Illumina’s EPIC methylation arrays. R package version 0.6.0 (2016).

[45] Zeilinger S, Kuhnel B, Klopp N, Baurecht H, Kleinschmidt A, Gieger C (2013). Tobacco smoking leads to extensive genome-wide changes in DNA methylation. PLoS One.

[46] Ambatipudi S, Cuenin C, Hernandez-Vargas H, Ghantous A, Le Calvez-Kelm F, Kaaks R (2016). Tobacco smoking-associated genome-wide DNA methylation changes in the EPIC study. Epigenomics.

[47] Monick MM, Beach SR, Plume J, Sears R, Gerrard M, Brody GH (2012). Coordinated changes in AHRR methylation in lymphoblasts and pulmonary macrophages from smokers. Am J Med Genet B Neuropsychiatr Genet.

[48] Lu AT, Quach A, Wilson JG, Reiner AP, Aviv A, Raj K (2019). DNA methylation GrimAge strongly predicts lifespan and healthspan. Aging.

[49] Logue MW, Miller MW, Wolf EJ, Huber BR, Morrison FG, Zhou Z (2020). An epigenome-wide association study of posttraumatic stress disorder in US veterans implicates several new DNA methylation loci. Clin Epigenetics.

[50] Smith AK, Ratanatharathorn A, Maihofer AX, Naviaux RK, Aiello AE, Amstadter AB (2020). Epigenome-wide meta-analysis of PTSD across 10 military and civilian cohorts identifies methylation changes in AHRR. Nat Commun.

[51] Cardenas A, Ecker S, Fadadu RP, Huen K, Orozco A, McEwen LM (2022). Epigenome-wide association study and epigenetic age acceleration associated with cigarette smoking among Costa Rican adults. Sci Rep.

[52] Sun YQ, Richmond RC, Suderman M, Min JL, Battram T, Flatberg A (2021). Assessing the role of genome-wide DNA methylation between smoking and risk of lung cancer using repeated measurements: the HUNT study. Int J Epidemiol.

[53] Christiansen C, Castillo-Fernandez JE, Domingo-Relloso A, Zhao W, El-Sayed Moustafa JS, Tsai PC (2021). Novel DNA methylation signatures of tobacco smoking with trans-ethnic effects. Clin Epigenetics.

[54] Boullon L, Abalo R, Llorente-Berzal A (2021). Cannabinoid Drugs-Related Neuroprotection as a Potential Therapeutic Tool Against Chemotherapy-Induced Cognitive Impairment. Front Pharmacol.

[55] Zhang L, Wang X, Yu W, Ying J, Fang P, Zheng Q (2022). CB2R Activation Regulates TFEB-Mediated Autophagy and Affects Lipid Metabolism and Inflammation of Astrocytes in POCD. Front Immunol.

[56] Campagna MP, Xavier A, Lechner-Scott J, Maltby V, Scott RJ, Butzkueven H (2021). Epigenome-wide association studies: current knowledge, strategies and recommendations. Clin Epigenetics.

[57] Wilson R, Wahl S, Pfeiffer L, Ward-Caviness CK, Kunze S, Kretschmer A (2017). The dynamics of smoking-related disturbed methylation: a two time-point study of methylation change in smokers, non-smokers and former smokers. BMC Genomics.

[58] McCartney DL, Stevenson AJ, Hillary RF, Walker RM, Bermingham ML, Morris SW (2018). Epigenetic signatures of starting and stopping smoking. EBioMedicine.

[59] Lucas CJ, Galettis P, Schneider J (2018). The pharmacokinetics and the pharmacodynamics of cannabinoids. Br J Clin Pharmacol.

[60] Komaki S, Ohmomo H, Hachiya T, Sutoh Y, Ono K, Furukawa R (2021). Longitudinal DNA methylation dynamics as a practical indicator in clinical epigenetics. Clin Epigenetics.

[61] Alshaarawy O. Total and differential white blood cell count in cannabis users: results from the cross-sectional National Health and Nutrition Examination Survey, 2005-2016. J Cannabis Res. 2019;1:1–7. 10.1186/s42238-019-0007-8 PMC7678768 33225221

[62] Abuhasira R, Haviv YS, Leiba M, Leiba A, Ryvo L, Novack V (2021). Cannabis is associated with blood pressure reduction in older adults—A 24-hours ambulatory blood pressure monitoring study. Eur J Intern Med.

[63] McPherson KL, Tomasi DG, Wang GJ, Manza P, Volkow ND (2021). Cannabis Affects Cerebellar Volume and Sleep Differently in Men and Women. Front Psychiatry.

[64] Solinas M, Ferre S, You ZB, Karcz-Kubicha M, Popoli P, Goldberg SR (2002). Caffeine induces dopamine and glutamate release in the shell of the nucleus accumbens. J Neurosci.

[65] Covey DP, Wenzel JM, Cheer JF (2015). Cannabinoid modulation of drug reward and the implications of marijuana legalization. Brain Res.

[66] Davies G, Lam M, Harris SE, Trampush JW, Luciano M, Hill WD (2018). Study of 300,486 individuals identifies 148 independent genetic loci influencing general cognitive function. Nat Commun.

[67] Walker RM, Christoforou AN, McCartney DL, Morris SW, Kennedy NA, Morten P (2016). DNA methylation in a Scottish family multiply affected by bipolar disorder and major depressive disorder. Clin Epigenetics.

[68] Lee JJ, Wedow R, Okbay A, Kong E, Maghzian O, Zacher M (2018). Gene discovery and polygenic prediction from a genome-wide association study of educational attainment in 1.1 million individuals. Nat Genet.

[69] Li M, Li Y, Qin H, Tubbs JD, Li M, Qiao C (2021). Genome-wide DNA methylation analysis of peripheral blood cells derived from patients with first-episode schizophrenia in the Chinese Han population. Mol Psychiatry.

[70] Chen MH, Raffield LM, Mousas A, Sakaue S, Huffman JE, Moscati A (2020). Trans-ethnic and Ancestry-Specific Blood-Cell Genetics in 746,667 Individuals from 5 Global Populations. Cell.

[71] Wu Y, Cao H, Baranova A, Huang H, Li S, Cai L (2020). Multi-trait analysis for genome-wide association study of five psychiatric disorders. Transl Psychiatry.

[72] Kim KW, Park SC, Cho HJ, Jang H, Park J, Shim HS (2021). Integrated genetic and epigenetic analyses uncover MSI2 association with allergic inflammation. J Allergy Clin Immunol.

[73] Goes FS, McGrath J, Avramopoulos D, Wolyniec P, Pirooznia M, Ruczinski I (2015). Genome-wide association study of schizophrenia in Ashkenazi Jews. Am J Med Genet B Neuropsychiatr Genet.

[74] Autism Spectrum Disorders Working Group of The Psychiatric Genomics C. (2017). Meta-analysis of GWAS of over 16,000 individuals with autism spectrum disorder highlights a novel locus at 10q24.32 and a significant overlap with schizophrenia. Mol Autism.

[75] Glossop JR, Emes RD, Nixon NB, Haworth KE, Packham JC, Dawes PT (2014). Genome-wide DNA methylation profiling in rheumatoid arthritis identifies disease-associated methylation changes that are distinct to individual T- and B-lymphocyte populations. Epigenetics.

[76] Ramer R, Bublitz K, Freimuth N, Merkord J, Rohde H, Haustein M (2012). Cannabidiol inhibits lung cancer cell invasion and metastasis via intercellular adhesion molecule-1. FASEB J.

[77] Seltzer ES, Watters AK, MacKenzie D, Jr., Granat LM, Zhang D. Cannabidiol (CBD) as a Promising Anti-Cancer Drug. Cancers (Basel). 2020;12:1–26. 10.3390/cancers12113203 PMC7693730 33143283

[78] Bloomfield MA, Ashok AH, Volkow ND, Howes OD (2016). The effects of Delta(9)-tetrahydrocannabinol on the dopamine system. Nature.

[79] Butovsky E, Juknat A, Elbaz J, Shabat-Simon M, Eilam R, Zangen A (2006). Chronic exposure to Delta9-tetrahydrocannabinol downregulates oxytocin and oxytocin-associated neurophysin in specific brain areas. Mol Cell Neurosci.

[80] Liu C, Sadat SH, Ebisumoto K, Sakai A, Panuganti BA, Ren S (2020). Cannabinoids Promote Progression of HPV-Positive Head and Neck Squamous Cell Carcinoma via p38 MAPK Activation. Clin Cancer Res.

[81] Marconi A, Di Forti M, Lewis CM, Murray RM, Vassos E (2016). Meta-analysis of the Association Between the Level of Cannabis Use and Risk of Psychosis. Schizophr Bull.

[82] Hjorthoj C, Posselt CM, Nordentoft M (2021). Development Over Time of the Population-Attributable Risk Fraction for Cannabis Use Disorder in Schizophrenia in Denmark. JAMA Psychiatry.

[83] Wittchen HU, Frohlich C, Behrendt S, Gunther A, Rehm J, Zimmermann P (2007). Cannabis use and cannabis use disorders and their relationship to mental disorders: a 10-year prospective-longitudinal community study in adolescents. Drug Alcohol Depend.

[84] Pinto JV, Medeiros LS, Santana da Rosa G, Santana de Oliveira CE, Crippa JAS, Passos IC (2019). The prevalence and clinical correlates of cannabis use and cannabis use disorder among patients with bipolar disorder: A systematic review with meta-analysis and meta-regression. Neurosci Biobehav Rev.

[85] Corsi DJ, Donelle J, Sucha E, Hawken S, Hsu H, El-Chaar D (2020). Maternal cannabis use in pregnancy and child neurodevelopmental outcomes. Nat Med.

[86] Di Forti M, Sallis H, Allegri F, Trotta A, Ferraro L, Stilo SA (2014). Daily use, especially of high-potency cannabis, drives the earlier onset of psychosis in cannabis users. Schizophr Bull.

[87] Secades-Villa R, Garcia-Rodriguez O, Jin CJ, Wang S, Blanco C (2015). Probability and predictors of the cannabis gateway effect: a national study. Int J Drug Policy.

[88] Blanco C, Hasin DS, Wall MM, Florez-Salamanca L, Hoertel N, Wang S (2016). Cannabis Use and Risk of Psychiatric Disorders: Prospective Evidence From a US National Longitudinal Study. JAMA Psychiatry.

[89] Hagenbach U, Luz S, Ghafoor N, Berger JM, Grotenhermen F, Brenneisen R (2007). The treatment of spasticity with Delta9-tetrahydrocannabinol in persons with spinal cord injury. Spinal Cord.

[90] Corey-Bloom J, Wolfson T, Gamst A, Jin S, Marcotte TD, Bentley H (2012). Smoked cannabis for spasticity in multiple sclerosis: a randomized, placebo-controlled trial. CMAJ.

[91] Funk AJ, McCullumsmith RE, Haroutunian V, Meador-Woodruff JH (2012). Abnormal activity of the MAPK- and cAMP-associated signaling pathways in frontal cortical areas in postmortem brain in schizophrenia. Neuropsychopharmacology.

[92] Howes OD, Kambeitz J, Kim E, Stahl D, Slifstein M, Abi-Dargham A (2012). The nature of dopamine dysfunction in schizophrenia and what this means for treatment. Arch Gen Psychiatry.

[93] Lee MR, Weerts EM (2016). Oxytocin for the treatment of drug and alcohol use disorders. Behav Pharmacol.

[94] Gravely S, Driezen P, Smith DM, Borland R, Lindblom EN, Hammond D (2020). International differences in patterns of cannabis use among adult cigarette smokers: Findings from the 2018 ITC Four Country Smoking and Vaping Survey. Int J Drug Policy.

[95] Auer R, Vittinghoff E, Yaffe K, Kunzi A, Kertesz SG, Levine DA (2016). Association Between Lifetime Marijuana Use and Cognitive Function in Middle Age: The Coronary Artery Risk Development in Young Adults (CARDIA) Study. JAMA Intern Med.

[96] National Academies of Sciences Engineering and Medicine (U.S.). Committee on the Health Effects of Marijuana: an Evidence Review and Research Agenda. The health effects of cannabis and cannabinoids: the current state of evidence and recommendations for research. : Washington, DC: The National Academies Press; 2017. xviii, 468. 28182367

