# Supplementary material for: Genome-wide DNA methylation association study of recent and cumulative marijuana use in middle aged adults
Source: Mol Psychiatry. 2023 May 31;28(6):2572–82. doi: 10.1038/s41380-023-02106-y (PMC10611566; doi:10.1038/s41380-023-02106-y)
Supplement: Supplementary file 1 — Supplemental Figures [file 41380_2023_2106_MOESM1_ESM.docx]

**Supplemental Figure 1.** Quantile-Quantile (Q-Q) plots of the -log_10_(*P* values) for recent and cumulative marijuana use at Y15 and Y20

1. Y15 Recent Marijuana Use B. Y15 Cumulative Marijuana Use

𝜆=1.05

𝜆=1.03

C. Y20 Recent Marijuana Use D. Y20 Cumulative Marijuana Use

𝜆=1.05

𝜆=1.08

The -log_10_(*P* values) are plotted against the corresponding expected -log_10_(*P* values).

**Supplemental Figure 2.** Venn diagram of significant CpGs associated with recent and cumulative marijuana use at Y15 and Y20

**
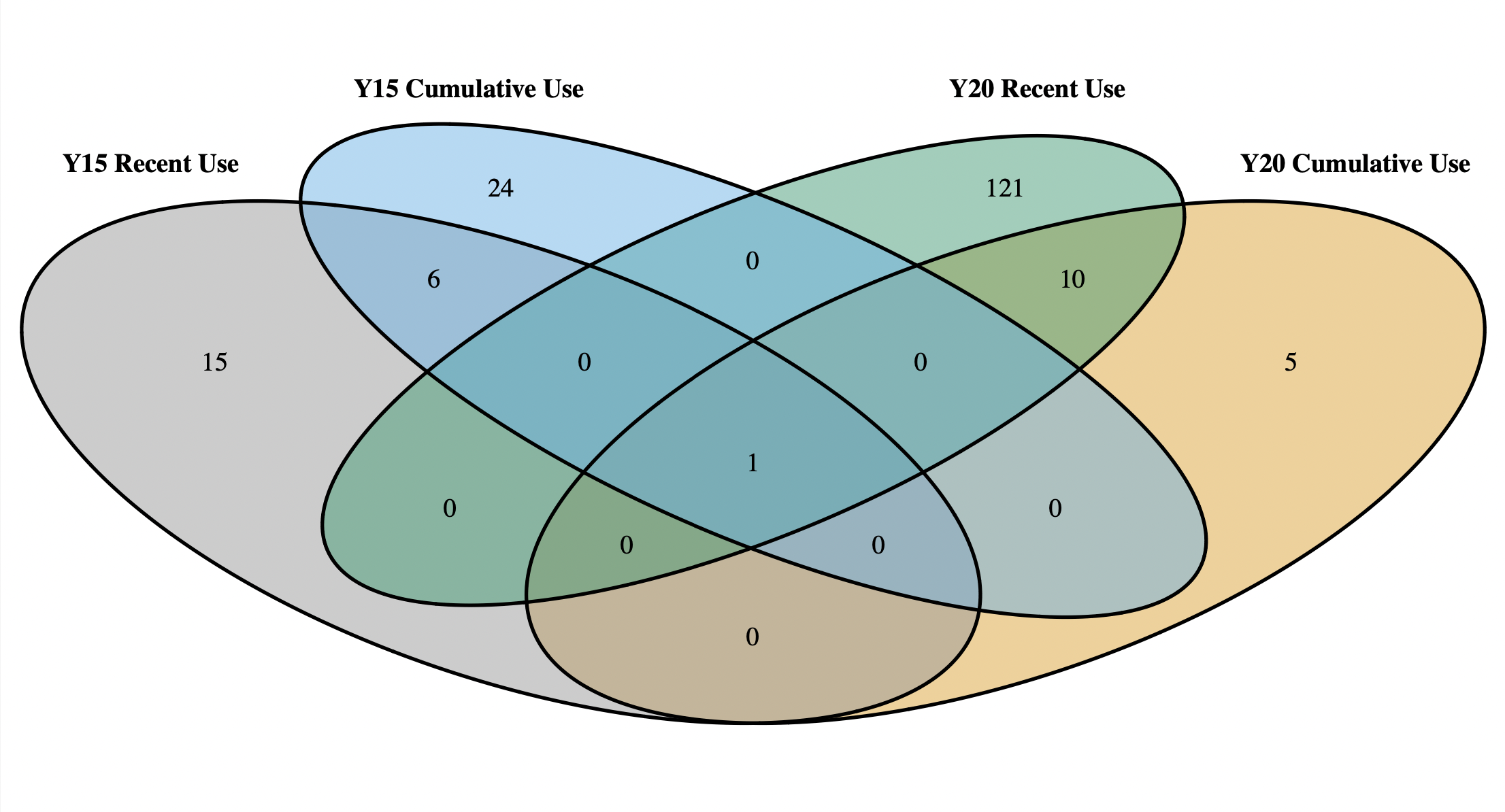
**

Supplemental Figure 3. Comparison of the beta coefficients between Δmarijuana use vs. Δmethylation analysis and marijuana use at Y15 and Y20

1. Recent Use

B. Cumulative Use

**Supplemental Figure 4.** Comparison of regression beta coefficients for identified recent and cumulative marijuana use CpGs at Y15 by sex

1. Recent Use

1. Cumulative Use

Bonferroni-corrected *P* values were used to determine significance (*P* < 0.05). Pearson’s correlation coefficient and *P* value are provided to assess the relationship of the regression coefficients for each comparison.

**Supplemental Figure 5.** Comparison of regression beta coefficients for identified recent and cumulative marijuana use CpGs at Y20 by sex

1. Recent Use

1. Cumulative Use

Bonferroni-corrected *P* values were used to determine significance (*P* < 0.05). Pearson’s correlation coefficient and *P* value are provided to assess the relationship of the regression coefficients for each comparison.

**Supplemental Figure 6.** Comparison of regression beta coefficients for identified recent and cumulative marijuana use CpGs at Y15 by self-reported race

1. Recent Use

1. Cumulative Use

Bonferroni-corrected *P* values were used to determine significance (*P* < 0.05). Pearson’s correlation coefficient and *P* value are provided to assess the relationship of the regression coefficients for each comparison.

**Supplemental Figure 7.** Comparison of regression beta coefficients for identified recent and cumulative marijuana use CpGs at Y20 by self-reported race

1. Recent Use

1. Cumulative Use

Bonferroni-corrected *P* values were used to determine significance (*P* < 0.05). Pearson’s correlation coefficient and *P* value are provided to assess the relationship of the regression coefficients for each comparison.

**Supplemental Figure 8.** Comparison of regression coefficients for identified recent marijuana use CpGs by smoking status at Y15

A) Non vs Former Smokers B) Non vs Current Smokers

C) Former vs Current Smokers

Bonferroni-corrected *P* values were used to determine significance (*P* < 0.05). Pearson’s correlation coefficient and *P* value are provided to assess the relationship of the regression coefficients for each comparison.

**Supplemental Figure 9.** Comparison of regression coefficients for identified cumulative marijuana use CpGs by smoking status at Y15

A) Non vs Former Smokers B) Non vs Current Smokers

C) Former vs Current Smokers

Bonferroni-corrected *P* values were used to determine significance (*P* < 0.05). Pearson’s correlation coefficient and *P* value are provided to assess the relationship of the regression coefficients for each comparison.

**Supplemental Figure 10.** Comparison of regression coefficients for identified recent marijuana use CpGs by smoking status at Y20

A) Non vs Former Smokers B) Non vs Current Smokers

C) Former vs Current Smokers

Bonferroni-corrected *P* values were used to determine significance (*P* < 0.05). Pearson’s correlation coefficient and *P* value are provided to assess the relationship of the regression coefficients for each comparison.

**Supplemental Figure 11.** Comparison of regression coefficients for identified cumulative marijuana use CpGs by smoking status at Y20

A) Non vs Former Smokers B) Non vs Current Smokers

C) Former vs Current Smokers

Bonferroni-corrected *P* values were used to determine significance (*P* < 0.05). Pearson’s correlation coefficient and *P* value are provided to assess the relationship of the regression coefficients for each comparison.
